# Interventional Pain Management for the Pediatric Cancer Patient: A Literature Review

**DOI:** 10.3390/children9030389

**Published:** 2022-03-10

**Authors:** Christina Le-Short, Kavya Katragadda, Neil Nagda, David Farris, Marianne Halphen Gelter

**Affiliations:** 1 Department of Pain Medicine, The University of Texas MD Anderson Cancer Center, Houston, TX 77030, USA; dpfarris@mdanderson.org (D.F.); mhgelter@mdanderson.org (M.H.G.); 2 Institute of Society and Genetics, The University of California, Los Angeles, CA 90095, USA; kkatragadda@g.ucla.edu; 3 Department of Anesthesiology, The University of Texas Health Science Center at Houston, Houston, TX 77030, USA; neil.nagda@uth.tmc.edu

**Keywords:** pediatric pain, cancer pain, pain interventions, interventional pain, pediatric cancer pain

## Abstract

This literature review examines the use of interventional treatments for pain management in pediatric cancer patients. While interventional procedures may be effective in cancer pain management, these procedures are infrequently employed in pediatric cohorts. This underutilization of interventional procedures may be supported by a deficit in randomized, controlled studies and literature regarding their role in pediatric cancer populations. Particularly because literature on the efficacy of interventional treatments in adult populations may not directly translate to pediatric populations, more research about the role of interventional procedures in managing pediatric cancer pain is necessary for a strengthened understanding of pediatric cancer pain treatment.

## 1. Introduction

Pain associated with pediatric cancer is a debilitating yet frequently reported symptom, from initial cancer diagnosis through end-of-life and survivorship [1]. Around 70% of children with cancer experience severe pain during their illness [2]. This pain can stem not only from the cancer itself but also from medical treatments and procedures [3]. The treatment of pediatric cancer pain is often complicated by difficulties in accurately assessing pain with pediatric patients and the higher treatment-response variability in children [4]. Traditionally, pediatric cancer pain management begins with analgesic drug usage in a two-step approach in accordance with pain intensity—from nonopioid analgesics for mild pain to opioid analgesics for moderate to severe pain [3].

Over 50% of pediatric cancer outpatients experience undertreated pain, which negatively affects patients’ emotional, psychological, and social well-being [5]. The severity of the consequences of insufficient pain management for a child’s quality of life has led to a greater consideration of multidisciplinary approaches to managing pediatric pain [6]. Interventional pain management techniques are often a component of these multidisciplinary approaches.

While numerous interventional techniques, such as nerve blocks, epidurals, and intrathecal therapies, are successfully employed in adult cancer pain management, these techniques are less commonly used in pediatric patient populations [7]. Whereas in adult cancer pain management—where intervention is often used early in the course of treatment—interventional techniques are often only considered in pediatric populations when all other treatment options fail [8]. Though interventional techniques may improve pain management for children experiencing cancer-associated pain, adult interventional pain management models may not be directly applicable to children [9]. Consequently, this literature review aims to investigate the efficacies of various interventional treatments for cancer pain in pediatric populations.

## 2. Literature Search

A medical research librarian (DF) searched MEDLINE (Ovid), Embase (Ovid), PubMed, and Scopus from inception to 13 August 2021. After consultation with the research team, the librarian developed and tailored the search strategy to each database and selected controlled vocabulary (MeSH and Emtree) and natural language terms for the concepts of cancer pain, children, and various treatment interventions. Searches were limited to English-only articles, but no other limiters or published search filters were used (Table 1).

We analyzed a total of 62 studies. We excluded 13 studies that were more than 10 years old to limit the scope to contemporary literature only. Of the remaining 49 studies, we excluded 20 that were focused on noninterventional pain management techniques only (medications or behavioral) and 6 studies on interventional pain management that did not include pediatric patients. Of the remaining 23 studies, 10 were case reports, 4 were retrospective studies, 3 were narrative reviews, 2 were nonrandomized prospective studies (both focused on osteoid osteomas), 3 were case series, and 1 was a cohort study (Table 2).

## 3. Discussion

### 3.1. Peripheral Nerve Blocks

Peripheral nerve blocks have been used in the treatment of various pediatric cancer pain conditions. While systemic analgesia is often used first in this population, it can be rendered ineffective or severely limited by adverse effects, including nausea, vomiting, pruritis, and sedation, which can be profound. Peripheral nerve blockade involves the delivery of local anesthetic medications, occasionally with adjuvants, such as alpha-2 agonists or corticosteroids, in proximity to a peripheral nerve to produce a targeted sensory block. This technique can be employed as a single injection, as repeated injections via a catheter, or as a continuous infusion [8]. A retrospective study involving 108 pediatric (age 2–18) patients undergoing orthopedic tumor surgery demonstrated the effectiveness of peripheral nerve blockade in this population [9]. The study included femoral, sciatic, axillary, supraclavicular, infraclavicular, and interscalene nerve blocks for surgically treated pathology, such as osteosarcoma, Ewing sarcoma, rhabdomyosarcoma, giant cell tumors, and other pediatric orthopedic tumors. The authors reported lower pain scores, longer duration to pain onset postoperatively, and lower total analgesic consumption in the population that received nerve blocks [10]. A case report depicted the success of an erector spinae plane block for analgesia in a 7-year-old patient undergoing resection of a large rib tumor [11]. 

Though these studies primarily examined the utility of peripheral nerve blocks in short-term, postoperative periods, similar efficacy may be seen in more chronic settings. A literature review of the use of regional anesthesia for pain management in pediatric palliative care described multiple successful cases of tunneled peripheral nerve catheters for long-term analgesia—up to 88 days in one case [12]. Potential complications from peripheral nerve blockade include bleeding, nerve injury, and local anesthetic systemic toxicity. However, the widespread use of ultrasonography mitigates these risks through the detection of relevant anatomy and direct visualization of local anesthetic spread. Recently, the phenomenon of rebound pain after regional anesthesia has been increasingly described in the literature. However, it is most often associated with regional anesthetics used to produce surgical anesthesia and not well described in chronic pain literature. One possible explanation could be that the use of corticosteroids in chronic pain procedures blunts the effect of rebound pain [13]. Evidently, peripheral nerve blockade can play a role in pain management for pediatric cancer patients, particularly those with tumors affecting the bones.

### 3.2. Sympathetic Blocks

Sympathetic blockade, commonly used to treat painful conditions, such as complex regional pain syndrome types 1 and 2, herpes zoster, diabetic peripheral neuropathy, and vascular insufficiency, has been applied in the treatment of pediatric cancer pain. Most commonly, celiac plexus blockade (CPB) is used to target visceral abdominal pain in the setting of upper gastrointestinal malignancies, such as pancreas, liver, gallbladder, spleen, stomach, and small intestine cancers. Because of its potential adverse effects—ranging from common complications, such as diarrhea and orthostatic hypotension, to rare, catastrophic risks, such as retroperitoneal hemorrhage and paraplegia—CPB is often limited to terminal patients in the pediatric oncologic population [8]. Typically, it is performed in patients with severe, intractable pain or profound adverse effects that limit the use of opioid therapy [14].Unfortunately, data on the use of CPBs in pediatric cancer are limited to a small number of cases. One such case detailed a 7-year-old patient with adrenal neuroblastoma and associated refractory abdominal pain who experienced adverse effects with opioids, including pruritus and sedation [14]. Another case described the similar use of celiac plexus neurolysis for abdominal pain in a 3-year-old patient with unresectable hepatoblastoma [14]. Both cases reported a reduction in previously reported adverse effects, a decrease in pain medication intake, and improvement in pain relief until the time of death, at approximately 3 months and 9 weeks, respectively [12]. A retrospective study of children and young adults with cancer demonstrated similar beneficial outcomes, including decreased opioid use and reduced pain scores, following CPB [14]. In one case, a 14-year-old patient with hepatoblastoma was able to discontinue opioid therapy for 6 months until his liver transplant. Another patient exhibited an initial reduction in morphine equivalent daily dose for 1 week postoperatively, followed by an escalation. The patient only survived for 16 days after the procedure, and dose escalation was attributed to disease progression [15]. Finally, Restrepo-Garces et al. [16] reported a case of pain reduction and quality-of-life improvement following ganglion impar neurolysis in a pediatric patient with perineal cancer-associated pain. Thus, the limited research that exists largely portrays favorable results and limited adverse effects, which may support earlier use of CPB in these patients. Potential complications arising from chemical neurolysis include necrosis of the skin and other non-target tissues, neuritis, anesthesia dolorosa, and prolonged motor paralysis. Another important consideration with these procedures involves the return of pain, and there is no consensus on the expected duration of pain relief due to confounding factors, such as progression of the underlying malignancy. Pain recurrence after denervation with a neurolytic is believed to be due to nerve regrowth.

Additional studies in this area are necessary to further elucidate the effectiveness of these interventions and other sympathetic blockade in pediatric cancer pain. 

### 3.3. Epidural Analgesia

Epidural analgesia is employed to decrease cancer-related pain secondary to tumor infiltration that is refractory to escalation in opioid therapy. Epidural analgesia may be performed either as a “single shot” or as a continuous epidural infusion of local anesthetic and/or opioid medication through a catheter. Catheter placement is often preferred, as it provides longer-term therapy. In pediatric patients, the catheter is often tunneled subcutaneously so as to avoid dislodgement during activity and decrease infection risk [8]. A 2013 review of regional anesthesia techniques used in pediatric palliative pain management describes several case studies of epidural analgesia use in patients suffering from neuroblastoma, astrocytoma, metastatic retinoblastoma, pelvic chondrosarcoma, and other pelvic and sacral masses [12]. Across all case studies reviewed, all patients were noted to have had satisfactory analgesia associated with the epidural [12]. 

Epidural analgesia can help to provide long-lasting relief to pediatric cancer patients; however, it is not without risk of complications. A 2018 case study of a 12-year-old patient with metastatic rhabdomyosarcoma-related pain described significant bleeding associated with the tunneling of the epidural catheter. The patient required transfusions of platelets, fresh frozen plasma, and packed red blood cells, and the bleeding stopped after the catheter was removed [17]. Other potential complications include accidental dural puncture with associated postdural puncture headache, catheter kinking, accidental dislodgement, or infection at the placement site. Despite these risks, epidural analgesia can be a safe and effective option for the treatment of cancer-related pain in children.

### 3.4. Intrathecal Therapies

Intrathecal therapies, such as catheters, implanted pain pumps, and neurolysis, can provide definitive pain relief for pediatric cancer patients and also allow for continued pain control once discharged from the hospital. Intrathecal catheters and implanted pain pumps allow for the continuous infusion of both opioids and local anesthetics, with some case reports describing the addition of such medications as clonidine and sufentanil for pediatric cancer pain [18,19]. Intrathecal infusions require exponentially smaller amounts of medication than do oral and intravenous routes, thereby decreasing the risk of opioid-induced side effects, such as severe constipation and sedation, which may negatively impact the patient’s quality of life [20,21]. Pumps can be titrated to effect, and many allow for the option of a patient-delivered bolus if deemed appropriate. An implanted intrathecal device allows the patient to participate in activities they would otherwise be precluded from due to fear of dislodging an external intrathecal or epidural catheter. 

In a prospective analysis of a large-scale, multicenter surveillance registry, intrathecal drug-delivery systems were shown to have a statistically significant benefit in cancer patients through reduction in pain scores and improvement in quality of life [22]. It is important to note, however, that the mean age in this analysis was 59 years. However, the minimum age was 13 years, and the analysis consisted of a large sample of 1403 patients. Furthermore, the authors did not find any statistically significant differences in these outcome measures among age groups [22]. A 2021 case study of a 16-year-old patient with terminal pelvic sarcoma described successful placement of an intrathecal pain pump delivering morphine [23]. The pump improved the patient’s quality of life and allowed her to enjoy activities, such as swimming, boating, painting, and games with friends, that she would not have been able to participate in otherwise [23]. 

Unfortunately, not all patients experience such a robust response. A 16-year-old patient with metastatic melanoma had an intrathecal pump placed to administer hydromorphone and bupivacaine; at 3 months after implantation, she noted her pain had only decreased from 9/10 to 7/10 [24]. Additionally, implant placement is not without risk. The implantation process is invasive, which puts an already immunocompromised cancer patient at risk for infection. Though rare, there is also the risk of pump error or failure. Despite these risks, intrathecal drug therapy should not be overlooked as an option for severe cancer pain in pediatric patients.

Intrathecal neurolysis is an option for refractory cancer pain and involves the application of chemical agents to target nerve fibers within the subarachnoid space, causing their degeneration and interrupting the transmission of pain signals. It can be an effective option for refractory pain in patients with a terminal diagnosis. There are only limited reports of intrathecal neurolysis in pediatric patients. One such report describes a 10-year-old patient with intractable pain secondary to recurrent right ilium osteosarcoma that was uncontrolled with maximum opioid therapy [25]. After intrathecal neurolysis of the L2-L5 levels, the patient’s pain was controlled with only a transdermal fentanyl patch, and she was able to spend time with her family at home before her passing. Intrathecal neurolysis is usually only considered when other options have been exhausted, as the procedure runs the risk of paralysis, paresis, and bowel and bladder dysfunction [25].

### 3.5. Cordotomy

Spinal cordotomy is an invasive intervention most commonly used in patients with uncontrollable pain in the setting of malignancy and a short life expectancy. It involves lesioning of the spinothalamic and spinoreticular tract to produce contralateral analgesia at 3 to 4 levels caudally. Owing to its potentially severe adverse effects, it is often performed after failure of traditional therapies, including systemic analgesia and minimally invasive procedures. The percutaneous approach is typically preferred over the open approach because it permits the use of electrical stimulation to verify electrode placement in the spinothalamic tract [26]. 

Steel et al. [27] reported two cases of open anterolateral cordotomy in pediatric cancer patients. The first was an 11-year-old patient with an inoperable right sciatic nerve sheath tumor that was refractory to chemotherapy. He experienced intractable pain that did not respond to either high-dose, multimodal analgesia or intrathecal analgesia with an opioid and a local anesthetic. After undergoing bilateral open thoracic anterolateral cordotomy, he experienced profound analgesia with eventual discontinuation of intrathecal and oral analgesics. The second case described a 10-year-old boy with caudal regression syndrome and a large, unresectable lipoma in the right gluteal and pelvic region. His pain was refractory to increasing doses of opioids and gabapentin, which caused substantial side effects. After left-sided cordotomy at T5, his pain and overall quality of life improved, and he was able to cease analgesic therapy [27]. Although the percutaneous approach is more feasible in adults than in children because patient cooperation is necessary to verify proper positioning through electrode stimulation, Reddy et al. [28] reported similar benefits with this approach in a pediatric patient with metastatic medulloblastoma. 

Neither of these case studies reported any major adverse complications resulting from the procedure. However, fatal complications have been associated with cordotomy, and fear of these complications may be the rationale behind their scarcity in clinical practice. Symptoms such as ataxia and paresis can result from unintentional lesions in the neighboring spinocerebellar and corticospinal tracts. Respiratory depression is a feared complication of the procedure at high cervical levels due to the close proximity of the reticulospinal tract, which contains fibers controlling spontaneous respiration. Postcordotomy pain, new neuropathic pain that often occurs contralaterally to the original pain, has also been reported to occur months after the procedure [26]. It is difficult to assess the prevalence and significance of these adverse effects due to the small sample of reported cases in the pediatric cancer population and the short lifespan of these patients. Nevertheless, spinal cordotomy has shown value in these subset of cases, and further research in the area may reinforce these findings.

### 3.6. Radiofrequency Ablation

Radiofrequency ablation (RFA) is a prevalent treatment modality in the management of various chronic pain conditions. It involves the application of an electric current to generate heat, which is used to ablate various structures, such as the nerves that innervate spinal facet joints. In the pediatric oncologic population, RFA is widely used in the management of osteoid osteoma. In these cases, pain is a predominant symptom that is often inadequately managed by long-term oral analgesics. Percutaneous RFA is a minimally invasive option that allows for destruction of the tumor with little impact to surrounding bone. A prospective, nonrandomized study involving 87 patients revealed the efficacy and safety of this treatment modality, with 75 patients reporting total pain relief and no analgesic requirement [29]. Nine additional patients underwent repeat RFA with similar results. Notably, the population in the study consisted of a wide age range from 3 to 55 years, and the average age was 14.5 [29]. In a retrospective study of 17 patients with osteoid osteoma, Arikan et al. [30] reported effective pain relief with percutaneous RFA. Another prospective study examined the safety of RFA and reported no major adverse effects; furthermore, as a secondary endpoint, efficacy was demonstrated, as none of the 21 patients reported recurrent pain during the 15.1-month mean follow-up period [31]. These studies also reported wide patient age ranges; however, the mean ages of both cohorts were relatively young (22 and 13.8, respectively), and the authors did not report any significant differences among age groups. 

Importantly, complications can be associated with RFA, such as thermal necrosis; infections, including cellulitis; and the radiation exposure itself. Chahal et al. [29] reported superficial skin infections in two patients, both of which promptly resolved with oral antibiotics. Ultimately, these studies depict RFA of osteoid osteoma as a safe and effective option for tumor treatment and pain in the pediatric population.

### 3.7. Vertebral Augmentation

Vertebral compression fractures are commonly associated with chemotherapy and in some patients can be a source of intractable pain. Balloon kyphoplasty has long been used as a treatment for compression fracture pain in adults. In the pediatric population, there are only a few reports of the use of balloon kyphoplasty. One report described the use of balloon kyphoplasty for the treatment of vertebral compression fractures in three children, two of whom had cancer [32]. The first patient was a 12-year-old boy with metastatic alveolar rhabdomyosarcoma and compression fractures of T8, T10, and T12, causing him 9/10 pain that was refractory to treatment [32]. Following balloon kyphoplasty, the patient reported his pain had decreased significantly to 2/10 while standing and 0/10 while sitting. The second patient was a 12-year-old boy with abdominal desmoplastic small round cell tumor and fractures at six levels: T4, T5, T6, T8, T9, and T10. Balloon kyphoplasty was performed in two stages, 1 week apart, after which the patient reported complete resolution of his pain. 

While there were no reports of complications in these case studies, rare complications of kyphoplasty include leakage of cement, pulmonary embolism, desaturation, and hypotension during cement application [32]. There is also some concern that the exothermic reaction the cement undergoes during hardening may have deleterious effects on the vertebral endplates’ potential for growth and remodeling [32]. Despite these concerns, this report shows that balloon kyphoplasty may be a safe option for treatment of pain in the pediatric cancer population. 

## 4. Conclusions

Interventional procedures for cancer pain in pediatric patients can be used as effective treatments for cancer-related pain in the pediatric population. Our review showed that the use of interventions was typically reserved for refractory pain that was not responsive to noninvasive treatment. This may show a bias towards conservatism in the pediatric population compared to the adult population, likely due to an increased fear of complications in children compared to adults. The majority of the literature we reviewed consisted of case reports, with only two nonrandomized, prospective studies, both of which focused on the treatment of osteoma-related pain. We realize that case reports may describe novel techniques and applications, but they are limited in their ability to provide any cause and effect relationships and are at risk of over-interpretation. Future randomized, controlled trials on interventional pain management in pediatric cancer patients are needed to determine their true efficacy in this population and potentially lead to improved pain outcomes in these children. 

## Figures and Tables

**Table 1:** Search strategies.

#	Search Terms	# Results from 13 August 2021
Database: Ovid MEDLINE^®^ALL 1946 to 13 August 2021		
1	Cancer Pain/	1832
2	((cancer or neoplasm or oncolog* or tumo?r) adj2 pain).ti,ab.	10,947
3	or/1–2 [Cancer Pain]	11,661
4	exp Child/	2,002,991
5	exp Infant/	1,185,178
6	Adolescent/	2,119,473
7	(child* or infant* or adolescen* or p?ediatric* or youth* or juvenile* or teen* or preschool or neonate* or newborn* or baby or babies or toddler* or boy or boys or girl*).ti,ab.	2,450,204
8	or/4–7 [Children]	4,426,168
9	3 and 8 [Cancer Pain + Children]	930
10	Cordotomy/	1269
11	exp Injections, Spinal/	16,668
12	exp Nerve Block/	23,998
13	Spinal Cord Stimulation/	1357
14	Radiofrequency Ablation/	1651
15	exp Vertebroplasty/	2925
16	(neurolysis or cordotom* or “nerve block*” or neuromodulation or “neuro-modulation” or “spinal cord stimulat*” or “peripheral nerve stimulat*” or “radiofrequency ablation” or “radio-frequency ablation” or “RFA therap*” or vertebroplasty or “vertebro-plasty” or kyphoplasty or ((intrathecal or “intra-thecal” or spinal or intraspinal or “intra-spinal”) adj (pump* or block* or injection*))).ti,ab.	53,469
17	or/10–16 [Procedures]	85,152
18	9 and 17 [Cancer Pain + Children + Procedures]	47
19	limit 18 to english language	37
Database: Embase Classic + Embase 1947 to August 13 2021		
1	Cancer Pain/	21,793
2	((cancer or neoplasm or oncolog* or tumo?r) adj2 pain).ti,ab.	18,791
3	or/1–2 [Cancer Pain]	30,401
4	exp Child/	3,195,813
5	Adolescent/	1,753,099
6	(child* or infant* or adolescen* or p?ediatric* or youth* or juvenile* or teen* or preschool or neonate* or newborn* or baby or babies or toddler* or boy or boys or girl*).ti,ab.	3,337,226
7	or/4–6 [Children]	4,947,658
8	3 and 7 [Cancer Pain + Children]	1989
9	Cordotomy/	2396
10	Neurolysis/	3620
11	Neuromodulation/	43,457
12	Spinal Cord Stimulation/	8050
13	Peripheral Nerve Stimulator/	450
14	Radiofrequency Ablation/	36,082
15	exp Percutaneous Vertebroplasty/	7664
16	(neurolysis or cordotom* or “nerve block*” or neuromodulation or “neuro-modulation” or “spinal cord stimulat*” or “peripheral nerve stimulat*” or “radiofrequency ablation*” or “radio-frequency ablation*” or “RFA therap*” or vertebroplasty or “vertebro-plasty” or kyphoplasty or “kypho-plasty” or ((intrathecal or “intra-thecal” or spinal or intraspinal or “intra-spinal”) adj (pump* or block* or injection*))).ti,ab.	84,681
17	or/9–16 [Procedures]	1,40,551
18	8 and 17 [Cancer Pain + Children + Procedures]	53
19	limit 18 to English language	48

**Table 2:** Studies included in this review *.

Procedure	Indications/Type of Cancer	Area of Body Treated	First Author and Year	Number of Cases **
Peripheral nerve block	Refractory pain, cancer-related pain, phantom limb pain, rib pain, neuralgia, CRPS, myofascial pain	Chest, upper limb, lower limb, shoulder	Argun 2019	108
			Muñoz 2017	1
Ganglion or plexus block	Refractory pain, cancer-related visceral pain	Abdomen, perineum	Anghelescu 2018	4
			Baek 2019	1
			Restrepo-Garces 2014	1
Epidural	Refractory pain, cancer pain/osteosarcoma	Lower extremities	Santana 2018	1
Intrathecal	Refractory pain, cancer pain/ osteosarcoma, rhabdomyosarcoma, Ewing-like sarcoma, metastatic melanoma, squamous cell carcinoma, neurofibromatosis type 1			
Intrathecal infusion		Lower extremity, bone, abdomen, perineum, pelvis	Mele 2021	1
			Conn 2020	2
			Bentley 2014	1
			Bengali 2014	1
			Higuchi 2017	1
			Whyte 2012	1
			Stearns 2020	1403 (ages reported: 13–93 y, median 59 y with no specific number of pediatric patients)
Intrathecal neurolysis		Esophagus, stomach pain	Tashiro 2021	1
Cordotomy	Unilateral refractory pain below the neck, cancer-related pain	Hip, abdomen, pelvis, knee	Steel 2017	2
			Reddy 2013	1
RFA/Cryoablation	Cancer pain, refractory pain	Femur, tibia, fibula, humerus, rib, acetabulum, ulna, calcaneus, iliac crest	Chahal 2017	87 (3–55 y, 14.5 y)
			Arikan 2016	17 (4–28 y, 13.8 y)
			Miyazaki 2016	21 (10–39 y, 22 y)
Kyphoplasty	Axial spine pain due to vertebral compression fracture/rhabdomyosarcoma, abdominal desmoplastic small cell tumor	Back/spine	Hoashi 2017	2

* Narrative reviews not included in this table due to lack of specified number of patients. ** Age range and mean age are provided if study was not limited to pediatric patients. Abbreviations: CRPS, complex regional pain syndrome; RFA, radiofrequency ablation.
